# Diagnosis and management of chagasic cardiomyopathy patients in several institutions in Argentina

**DOI:** 10.3389/fpara.2023.1195646

**Published:** 2023-07-24

**Authors:** Roberto Chuit, Laura Antonietti, Roberto Nicolás Agüero, Gabriela Badino Varela, Oscar Daniel Mordini, Emilce Alemandri, Marcelo Abril, Miguel Días, Zaida E. Yadón, Hugo Pizzi, Rogelio Pizzi

**Affiliations:** ^1^ Instituto de Investigaciones Epidemiológicas, Academia Nacional de Medicina, Buenos Aires, Argentina; ^2^ Fundación Mundo Sano, Buenos Aires, Argentina; ^3^ Hospital Rawson, Ministerio de Salud, Cordoba, Argentina; ^4^ Facultad de Ciencias Medicas de Cordoba, Universidad Nacional de Cordoba, Cordoba, Argentina

**Keywords:** Chagas disease, cardiomyopathy, diagnosis and management, heart failure, Argentina

## Abstract

**Introduction:**

According to estimates by the World Health Organization, the infection and disease caused by the protozoan parasite *Trypanosoma cruzi* affects almost 6 million people, and more than 1 million suffer chagasic cardiomyopathy (Ch-CMP). It is estimated that 376,000 of these individuals live in Argentina. This study describes the characteristics and medical management of individuals with Ch-CMP in Argentina.

**Methods:**

This is a descriptive, retrospective, cross-sectional study on the diagnosis and clinical and therapeutic evaluation of patients with Ch-MCP using historical records collected from different medical institutions in the country between 1 January 2018 and 30 June 2021.

**Results:**

During this period, 652 patients (mean age 61.2 years ± 12.9) were included, with women accounting for 60.3% of the sample. The diagnosis of cardiac insufficiency was 36.0% and 64.4% had arrhythmias. The most common cardiovascular risk factors detected were arterial hypertension (69.5%), smoking (56.6%), and diabetes (20.9%). Less than half of the subjects (45.4%) had been studied by electrocardiogram (ECG), chest X-ray, and echocardiogram. ECG studies showed conduction disorders (38.8%), left ventricular hypertrophy (28.1%), ventricular extrasystoles (22.0%), complete right bundle branch block (8.6%), and atrioventricular block (2.6%). According to the Kuschnir classification, 21.4% of the study subjects were in Grade 3.

**Conclusions:**

The patients included in the study had a similar clinical presentation and history of the disease to those published in other studies. When evaluating the medical practices, we found that patients were inadequately studied. Although it is difficult to estimate the fraction of the total number of patients represented by the present study, the study allowed us to establish that the care received by patients was not adequate.

## 1 Introduction

Chagas disease (ChD), or American trypanosomiasis, is a parasitic disease originally from the Americas, caused by *Trypanosoma cruzi* and described by Carlos Chagas at the beginning of the 20th century (Coura and Borges-Pereira, 2010). The first reports of cardiac manifestations and/or alterations were recorded by Salvador Mazza in 1926 (Mazza and Jorg, 1936), which included hypotension and splitting of the second heart sound, and were reinforced in the mid-1950s (Rosenbaum, 1964; Pinto Dias, 1982).

The World Health Organization (WHO) estimates that *T. cruzi* infection and illness affects almost 6 million people (Chagas disease in Latin America: an epidemiological update based on 2010 estimates). Over 1 million of these individuals suffer chagasic cardiomyopathy (Ch-CMP), and 376,000 live in Argentina.

ChD is endemic in areas of Latin America that were initially associated with vector transmission. Nonetheless, in recent decades, the disease’s detection has increased in non-endemic regions. It requires medical attention, mainly because of population migratory processes (Schmunis and Yadon, 2010).

When infection occurs, the acute stage of the disease is usually asymptomatic or oligosymptomatic. In most cases, the chronic phase evolves into an indeterminate form, characterized by positive serology without evidence of organic involvement; people who develop clinical manifestations are usually affected by myocardial disease, digestive disease, or both (Ribeiro et al., 2012). Chagasic cardiomyopathy (Ch-CMP) is characterized by progression toward myocardial compromise, which may present with normal ECG findings, minimal electrocardiographic (Pinto Dias, 1985) alterations, or even dilated cardiac chambers with a general deterioration of ventricular function, manifesting as cardiac insufficiency (Marin Neto et al., 2013; Marín-Neto et al., 2015; Nunes et al., 2018).

The disease’s leading clinical and organic manifestation is cardiac and is associated with premature cardiovascular morbi-mortality, mainly by sudden death, followed by cardiac insufficiency and thromboembolism (Biolo et al., 2010; Rassi et al., 2010). Although there are regional variations (Martins-Melo and Heukelbach, 2013), in Argentina, it is estimated that 1% of patients with Ch-CMP will die from cardiac insufficiency (Manzullo and Chuit, 1999; Viotti et al., 2006). The factors associated with an increased risk of the development of myocardiopathy are still not completely understood (Elizari, 1999). There is debate surrounding if the parasite has direct involvement in causing a direct lesion (Bellotti et al., 1996), an autoimmune phenomenon (Takle and Hudson, 1989), or mixed mechanisms with microvascular alterations and autonomic denervation (Dávila-Spinetti et al., 2005). Therefore, it is possible to estimate that the organic manifestation of the disease is the result of multifactorial processes (Medina-Rincón et al., 2021).

Moreover, although the prevention and control of ChD, as well as a general improvement in quality of life, has contributed to a decrease in the incidence of the disease, there is still a deficit in access to and the quality of medical care for chagasic patients. Approximately 10% of the individuals infected with *T. cruzi* have access to early diagnosis, and only 1% have access to timely and adequate treatment (Medina-Rincón et al., 2021). Among the causes of this phenomenon are a lack of specific knowledge from the health personnel; the silent nature of the disease, which affects mainly rural populations; and barriers to health services for the patients (Fanjul et al., 2016).

This study aims to describe the characteristics of a cohort of patients with Ch-CMP and the medical attention they received in institutions providing low and medium levels of medical care across diverse regions of Argentina. The objective is to provide knowledge and evidence that can be used in decision-making processes to support prompt diagnosis, follow-up, and treatment.

## 2 Methods

A cross-sectional, descriptive, and retrospective study of hospital-based medical practices (diagnosis, clinical evaluation, and treatment) was performed for individuals diagnosed with Ch-CMP, whose information was collected through a network of professionals coordinated by the Institute of Epidemiological Research, National Academy of Medicine of Buenos Aires. The data were collected through a specifically designed structured questionnaire that was accessible through the internet (web based).

The data collected were obtained from the medical records of patients who received on-site or off-site diagnostic tests and complementary studies from low- and medium-capability institutions, such as an electrocardiogram, radiology, ultrasound, a stress exercise test, and a Holter monitor. The historical data collected were obtained from records of patients that received medical attention between January 2018 and June 2021, provided by professionals from various states of Argentina (Chaco, Córdoba, Corrientes, Formosa, Salta, Jujuy, Tucumán, Ciudad de Buenos Aires, and Buenos Aires) associated with the coordinating institution. As the study focused on the medical care received, the facilities and areas of Argentina where they were located were undifferentiated.

The inclusion criteria in the current study were patients ≥ 18 years of age with a recorded diagnosis of ChD infection as outlined by national (Enfermedades Infecciosas, 2018) and international guidelines (Cucunubá et al., 2017; Pan American Health Organization (2019) (two positive serologic tests) and with a myocardiopathy diagnosis.

Ch-CMP was defined by the existence of one or more of the following alterations: a) ECG disorder: complete blockage of right bundle branch (RBBB); Left anterior fascicular block (LAFB); RBBB + LAFB; first degree atrioventricular (AV) block or greater; atrial flutter (AF) /atrial fibrillation (AF); ventricular extrasystoles (VE); or b) ECG disorders: systolic diameter of the left ventricle > 55 mm; ejection fraction (EFy) < 50%; ventricular aneurysm or apical or posterior-basal segmental wall motion informed as moderate to severe in the history report file.

Tobacco consumption, alcohol consumption, hypertension, diabetes, dyslipidemia, and other pathologies obtained from clinical records from the medical institution’s history file were registered.

To determine the functional capacity of the heart, the records were stratified according to the Kuschnir classification (Kuschnir et al., 1985), which establishes four stages (0 to 3) based on the evaluation of complementary basic tests and diagnosis of cardiac insufficiency.

## 3 Results

Of 1,150 records analyzed, 652 patients fulfilled the inclusion criteria, presenting positive serologic tests for *T. cruzi* and Ch-CMP. The mean age of cases was 61.2 years (SD ± 12.9). In addition, 393 (60.3%) were female and 259 (39.7%) male.

The most prevalent cardiovascular risk factors were hypertension (*n* = 453; 69.5%), smoking (*n* = 369; 56.6%), dyslipidemias (*n*= 248: 38,1%), and diabetes (*n* = 136; 20,9%). (**Table 1**).

**Table 1:** Prior pathological history of patients with chagasic cardiomyopathy (Ch-CMP).

Condition	n	%
Factors for cardiovascular risk		
Arterial hypertension	453	69.5
Smoking	369	56.6
Dyslipidemia	248	38.1
Diabetes	136	20.9
Prior pathological history		
Chronic obstructive pulmonary disease (COPD)	74	11.3
Chronic renal insufficiency	65	10.0
Chronic liver disease	53	8.1
Cerebrovascular accident (CVA)/Transient ischemic attack (TIA)	25	3.8

Cohort of 652 patients in Argentina between January 2018 and June 2021.

Source: Institute of Epidemiological Research, National Academy of Science of Buenos Aires.

**Figure 1:**
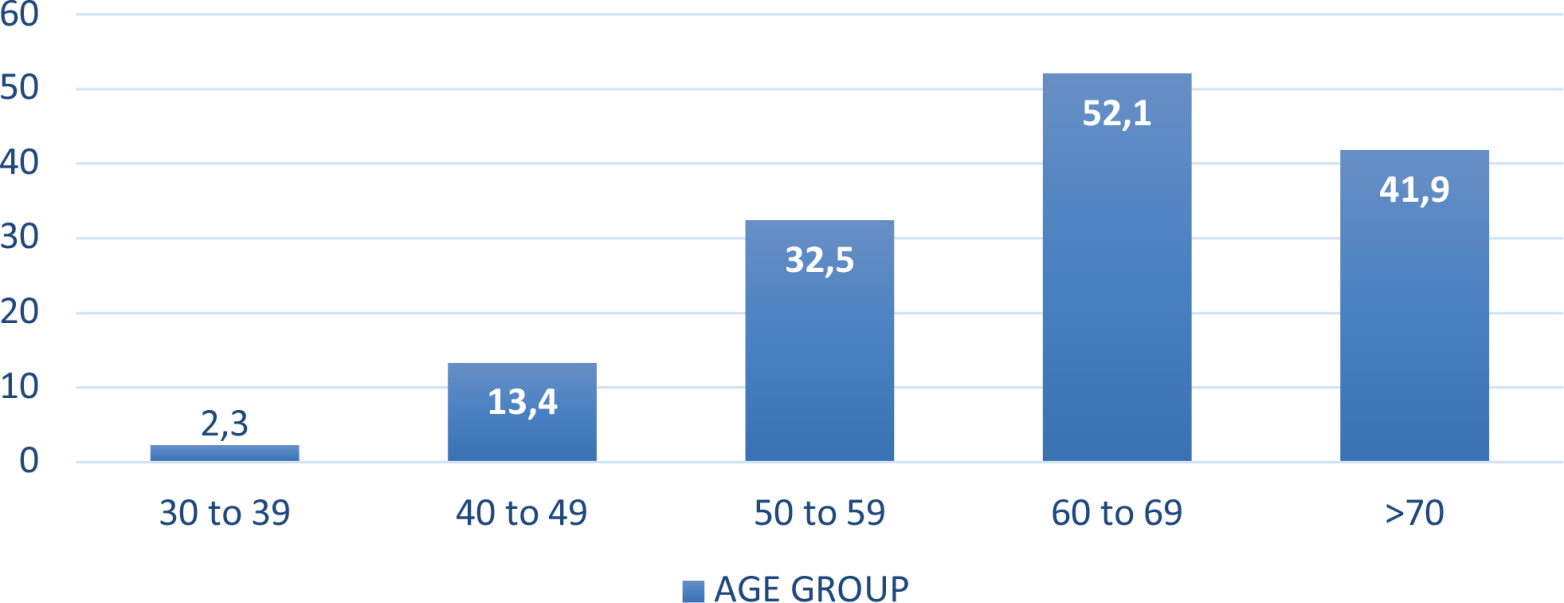
Distribution of cardiac insufficiency (CI) by age group in patients with chagasic cardiomyopathy (Ch-CMP). Cohort of 652 patients in Argentina between January 2018 and June 2021. *Source*: Institute of Epidemiological Research, National Academy of Medicine of Buenos Aires.

The distribution of cardiac insufficiency (CI) in patients with chagasic cardiomyopathy (Ch-CMP) by age group (n = 235) is presented in **Figure 1**. Although this condition affected individuals ranging from 30 years of age to older than 80 years of age, incidence increased with age and was higher in those older than 55 years, who represented 79.9% of the study population and 95.7% of the cases with CI.

Of 420 patients presented with arrhythmias, 128 (30.5%) presented with ventricular arrhythmias and 85 (20.2%) presented with fibrillation or atrial flutter. The type of arrhythmia was not specified in 207 (49.3%) patients. The history of cardiac device implantations was recorded in only 32 patients older than 50 years.

Medical practices performed on these patients are shown in **Table 2**. ECG (93.3%) was the most performed medical exam, followed by a thoracic X-ray (73.6%), stress exercise test (SET) (70.1%), echocardiogram (46.0%), and Holter monitor (38.2%). Less than half of the patients (n = 296; 45.4%) have records for ECG, chest X-Ray, and echocardiogram.

**Table 2:** Distribution of diagnostic tests reported in patients with chagasic cardiomyopathy (Ch-CMP) by age.

Age range	n	Electrocardiogram (ECG)		Stress exercise test (SET)		Holter monitor		Chest X-ray		Echocardiogram	
		n	%	n	%	n	%	n	%	n	%
30–34	10	9	90.0	7	70.0	4	40.0	6	60.0	4	40.0
35–39	34	32	94.1	27	79.4	10	29.4	22	64.7	18	52.9
40–44	33	30	90.9	24	72.7	13	39.4	26	78.8	13	39.4
45–49	34	32	94.1	24	70.6	9	26.5	25	73.5	19	55.9
50–54	96	84	87.5	54	56.2	34	35.4	60	62.5	51	53.1
55–59	101	97	96.0	74	73.3	35	34.7	66	65.3	48	47.5
60–64	123	123	100.0	103	83.7	42	34.1	113	91.9	58	47.2
65–69	46	38	82.6	21	45.7	13	28.3	30	65.2	17	37.0
70–74	102	90	88.2	61	59.8	39	38.2	66	64.7	40	39.2
75–79	38	38	100.0	33	86.8	22	57.9	36	94.7	19	50.0
> 80	35	35	100.0	29	82.9	28	80.0	30	85.7	13	37.1
**Total**	**652**	**608**	**93.3**	**457**	**70.1**	**249**	**38.2**	**480**	**73.6**	**300**	**46.0**

Cohort of 652 patients in Argentina between January 2018 and June 2021.

Source: Institute of Epidemiological Research, National Academy of Science of Buenos Aires.

In 608 patients, 1,254 electrocardiographic alterations were recorded: 408 (38.8%) patients presented with unspecific disorders of conduction, 352 (28.1%) with left ventricular hypertrophy, 276 (22.0%) with ventricular extrasystole, 108 (8.6%) with complete right bundle branch block, and 32 (2.6%) with atrioventricular block. The chest X-Ray performed on 480 individuals detected an increase in the cardiac silhouette in 334 individuals (69.6%). The SET was performed in 457 individuals, with most of the results showing no alterations (n = 387; 84.6%) and 70 (15.4%) patients presenting with an abnormal result. The most common alterations were related to repolarization disorders (n = 38; 54.3%), followed by ventricular arrhythmias (n = 18; 25.7%), isolated supraventricular arrhythmias (n = 7; 1.0%), and frequent ventricular extrasystole during maximum stress (n = 7; 1.0%).

In total, 249 24-hour Holter ECGs were performed, of which 116 (46.6%) presented alterations: 90 (77.6%) with ventricular extrasystole, 23 (19.8%) with supraventricular and extrasystole, and 3 (2.6%) with ventricular tachycardia. In addition, 23 (19.8%) records were associated with > 2-second pauses.

Radio-isotopic tests were performed in only 16 (2.5%) registered patients: four (25.0%) had severe anterolateral ischemia, ventricular dilation, and deterioration of the ventricular function, while nine (56.3%) did not have any evidence of pathology. In four (25.0%) patients, dilation of the left ventricular cavity and severe global hypokinesia were recorded.

When analyzing registered treatments, specific antiparasitic treatment (without differentiation between benznidazole and nifurtimox) was given to 39 (6.0%) patients, aldosterone antagonists to 311 (47.7%) patients, angiotensin-converting enzyme inhibitors to 275 (42.2%) patients, beta-blockers to 181 (27.8%) patients, angiotensin II receptor antagonists (AIIRA) to 178 (27.3%) patients, diuretics to 116 (17,8%) patients, and only one of those registered had amiodarone as an indication

Concerning the reported hospital admissions from June 2020 to June 2021, 103 (15.8%) patients were admitted due to ChD. Some of these patients were admitted only once (n = 23; 22.3%), some were admitted twice (n = 68; 66.0%), and 12 (11,7%) had three or more admissions. The age range of individuals with the highest need for hospital admission was between 50 and 69 years old.

To determine the functional capacity of the heart in this cohort of patients, the records were distributed according to the Kuschnir classification. A total number of 504 patients could be classified as follows (**Table 3**): 181 (35.9%) as Grade 1, 215 (42.7%) as Grade 2, and 108 (21.4%) as Grade 3. Of those in Grade 3, 33 (30.6%) required one or more admissions in the last year.

**Table 3:** Distribution of patients with heart disease according to the Kuschnir classification.

No. of hospital admissions	Kuschnir Classification					
	Grade 1		Grade 2		Grade 3	
	(n = 181)		(n = 215)		(n = 108)	
	n	%	n	%	n	%
1	1	0.55	12	5.6	3	2.8
2			42	19.5	26	24.1
3			8	3.7	4	3.7
Total	1	0.55	62	28.8	33	30.6

Cohort of 504 patients in Argentina between June 2020 and June 2021.

Source: Institute of Epidemiological Research, National Academy of Science of Buenos Aires.

## 4 Discussion

The current study describes the clinical and epidemiological characteristics of and the medical attention received by a cohort of patients with Ch-CMP from historical clinical records from Argentina.

Data from these records show that women (60.3%) seek medical attention more often than men, which may be explained by cultural, labor, or social factors (Kuschnir et al., 1985; Rosa-Jiménez et al., 2005). This should be studied in greater depth, although a recent study showed between-sex differences, with men having a higher incidence of myocardial fibrosis and worse ventricular remodeling than women (Assunção et al., 2016).

The average age of patients was in line with other hospital-based studies, where advancing age is one of the established risk factors for progression to Ch-MCP, as shown by our research and analyzed by other authors (Echalar et al., 2021).

In the analysis of risk factors and comorbidities, our study shows similar results to those registered in other studies of individuals with PMC. (Freitas Lidani et al., 2020; Cutshaw et al., 2023). Hypertension was observed in 69.5% of the patients in this study, a similar percentage to that found in the CONAREC XII registry (Perna et al., 2015; Assunção et al., 2016), where 69.3% had heart failure. Diabetes was recorded in 20.9% of patients, slightly lower than that found by RETIC (Instituto Nacional de Estadística y Censos - I.N.D.E.C, 2019). Population studies (Menéndez et al., 2006; Slimel et al., 2010; Corradi et al., 2012) report values lower than those found here, probably due to a selection bias, given the characteristics of the cohort.

Heart failure was diagnosed in 36.0% of the cohort, which is expected in this population (Delucchi et al., 2017); 21.4% of the patients were classified as Kuschnir Grade 3 and 30.6% of this group required at least one hospitalization in the last year. The classification allows for an estimate of the severity of myocardial compromise and, consequently, the need for medical attention. If carried out adequately, this will improve quality of life and reduce costs of care.

The WHO estimates that more than 376,000 people in Argentina require medical attention due to ChD in health institutions across the country and particularly in patients with CMP associated with *T. cruzi* infection (Chagas disease in Latin America: an epidemiological update based on 2010 estimates). In Argentina, national guidelines (Enfermedades Infecciosas, 2018) establish that every infected person in the chronic stage of the disease must have at least an electrocardiogram exam, chest X-ray, and/or electrocardiogram study, as well as an annual evaluation of their health status. Surprisingly, in this study cohort, half of the patients with Ch-CMP were not evaluated according to the minimum requirements established by the guidelines based on the scientific consensus. Non-compliance with the guidelines, which makes it possible to establish functional capacity, and the possible risk, which makes it possible to schedule medical care adjusted to the person's need, indicates a low quality of care.caring for patients with Ch-CMP

In cases with pathological evidence, different scientific societies (Sociedad Argentina de Cardiologíá, 2019; Sociedad Sudamericana de Cardiologí|á, 2019) recommend performing other cardiological studies that evaluate the degree of cardiac structural compromise and functional capacity.

One of the strengths of this study is its comprehensive national coverage, given the participation of professionals from various regions of the country, and therefore “real-life” evidence was obtained. The low proportion of recommended diagnostic tests and treatments recommended to patients with myocardial compromise demonstrates the low adherence to expert recommendations.

## 5 Conclusion

The current study of individuals infected with *T. cruzi* and presenting with associated cardiomyopathy and other related comorbidities shows similar results to those found in other studies. With the objective of determining the medical practices performed on these types of patients, we found that Ch-CMP receives insufficient and inadequate care from public and private health systems, which are non-compliant with national guidelines (Sociedad Argentina de Cardiologíá, 2019; Sociedad Sudamericana de Cardiologí|á, 2019; Enfermedades Infecciosas, 2018) and the scientific consensus.

It is crucial to continue to amplify the study network; promote educational programs for data registry, collection, and analysis; and urgently address the suboptimal medical attention received by those with Ch-CMP by aiming for better compliance with and access to the healthcare practices and interventions established by evidence-based recommendations.

Our study is subject to limitations, mainly because it is a retrospective descriptive study using secondary data that may lack quality and completeness. In many of the complementary studies we did not have access to the original records, only transcribed reports in the medical records. In addition, we cannot ensure that the study sample is representative of the population of patients with CD in Argentina since it was not randomly selected and was limited only to the data collected in the centers that participate in the network of professionals coordinated by the Institute of Epidemiological Investigations of the National Academy of Medicine of Buenos Aires. Having established this, we consider that the information provided allows us to believe that a better quality of care for patients with Ch-CMP is necessary, as is improving the education and communication skills of health teams and ensuring adequate access to medical care for patients with Ch-CMP.

Further studies are ongoing using our database, and we hope that results might provide more detailed information on this population.

## Data availability statement

The raw data supporting the conclusions of this article will be made available by the authors, without undue reservation.

## Ethics statement

This study respected ethical principles and current regulatory norms; it was approved by the ethical committee of the National Academy of Medicine (Act 12906/18/E). The participation of the professionals was voluntary and expressed through digital informed consent. Data were extracted from the clinical history of the patients by the professionals in an anonymous manner and automatically coded without temporal relation to the medical attention or analysis. The data were registered in a specifically designed web form that did not require the input of personal data. The inclusion of data did not imply, in any case, the modification of clinical conduct since there was no temporal relation.

## Author contributions

The data were collected and the manuscripts was written, read, and approved by all authors.
